# Rebelling against the (Insulin) Resistance: A Review of the Proposed Insulin-Sensitizing Actions of Soybeans, Chickpeas, and Their Bioactive Compounds

**DOI:** 10.3390/nu10040434

**Published:** 2018-03-30

**Authors:** Jaime L. Clark, Carla G. Taylor, Peter Zahradka

**Affiliations:** 1 Canadian Centre for Agri-Food Research in Health and Medicine, St. Boniface Hospital Albrechtsen Research Centre, Winnipeg, MB R2H 2A6, Canada; jclark@sbrc.ca (J.L.C.); ctaylor@sbrc.ca (C.G.T.); 2 Department of Food and Human Nutritional Sciences, University of Manitoba, Winnipeg, MB R3T 2N2, Canada; 3 Department of Physiology and Pathophysiology, University of Manitoba, Winnipeg, MB R3E 0T5, Canada

**Keywords:** soybeans, pulses, chickpeas, insulin resistance, bioactive compounds, GLUT4, PPARγ, adipokines, adiponectin, short-chain fatty acids

## Abstract

Insulin resistance is a major risk factor for diseases such as type 2 diabetes and metabolic syndrome. Current methods for management of insulin resistance include pharmacological therapies and lifestyle modifications. Several clinical studies have shown that leguminous plants such as soybeans and pulses (dried beans, dried peas, chickpeas, lentils) are able to reduce insulin resistance and related type 2 diabetes parameters. However, to date, no one has summarized the evidence supporting a mechanism of action for soybeans and pulses that explains their ability to lower insulin resistance. While it is commonly assumed that the biological activities of soybeans and pulses are due to their antioxidant activities, these bioactive compounds may operate independent of their antioxidant properties and, thus, their ability to potentially improve insulin sensitivity via alternative mechanisms needs to be acknowledged. Based on published studies using in vivo and in vitro models representing insulin resistant states, the proposed mechanisms of action for insulin-sensitizing actions of soybeans, chickpeas, and their bioactive compounds include increasing glucose transporter-4 levels, inhibiting adipogenesis by down-regulating peroxisome proliferator-activated receptor-γ, reducing adiposity, positively affecting adipokines, and increasing short-chain fatty acid-producing bacteria in the gut. Therefore, this review will discuss the current evidence surrounding the proposed mechanisms of action for soybeans and certain pulses, and their bioactive compounds, to effectively reduce insulin resistance.

## 1. Introduction

It is well known that insulin resistance is a condition that lays the foundation for diseases such as metabolic syndrome, type 2 diabetes, and cardiovascular disease [1]. As the precursor to the onset of overt disease, insulin resistance is characterized by a reduced cellular response to insulin and this requires the body to compensate by increasing insulin secretion to obtain the biological effects normally achieved with a lower amount of insulin [2,3]. While the exact cause of insulin resistance is unknown, there are multiple factors that can contribute to the reduced insulin sensitivity of target cells, including genetic and lifestyle factors [4].

Current therapeutic strategies for attenuating insulin resistance aim to encourage lifestyle modifications (e.g., diet, exercise, weight loss) as a first-line offense prior to the administration of pharmacological agents (e.g., insulin-sensitizing drugs) to patients [5]. The goal of pharmacotherapy in this disease context is to restore the normal relationship between insulin sensitivity and secretion [6]. However, due to the contrary complications of insulin-sensitizer drugs, alternative remedies for attenuating insulin resistance in the form of dietary agents are receiving more interest among patients and practitioners [7].

Legumes are an integral part of a healthy diet as they are rich in fibre, protein, complex carbohydrates and micronutrients, and contain no cholesterol [8,9]. In addition to their nutritional benefits, most legumes contain a variety of bioactive compounds that may add to their functional health benefits [10]. Legumes such as soybeans and pulses are known to be beneficial for diabetes management due to their low glycemic index [11,12], defined as producing a relatively low rise in blood glucose following their consumption [13]. Soybeans and pulses are both members of the Leguminosae family; however, the soybeans are grouped separately [12]. Both groups are nitrogen-fixing, meaning they produce their own nitrogen compounds that are released into the soil, thus eliminating the need for chemical fertilizers [14]. The primary characteristic that separates soybeans from pulses is that soybeans are oil-producing plants commonly harvested for the purpose of producing soybean oil, while pulses (dried beans, dried peas, chickpeas, lentils) are harvested dry as edible seeds [12]. Both are important sources of plant-based dietary protein world-wide [15,16]; in Asian countries, soybeans, in particular, are the major protein source for over one billion people [16]. In addition to their diabetes management properties, many clinical studies support the concept that consuming soybeans and pulses is favorable as a result of the ability to attenuate insulin resistance as shown by improvements in the homeostasis modelling assessment—insulin resistance (HOMA-IR) index and fasting insulin levels (Table 1) [5,17,18,19,20,21,22,23,24,25,26], although some studies have reported opposing [24] or null effects [25,27,28,29]. To this point, however, no one has summarized the evidence supporting the proposed mechanisms of action for soybeans and pulses in the context of insulin resistance. To address this gap in knowledge, this review will discuss the current evidence surrounding the proposed mechanisms of action for soybeans, certain pulses, and their bioactive compounds, to effectively reduce insulin resistance. 

## 2. The Search Strategy Used for This Review

The intent of this review was to summarize the information pertaining to soybeans and the four major pulses grown in North America (dried beans, dried peas, chickpeas, lentils) with respect to their proposed insulin-sensitizing actions. However, based on the literature search, most of the studies were on soybeans and chickpeas, with limited information available for dried beans, dried peas, and lentils. The literature search was conducted using the PubMed database through the University of Manitoba library with combinations of the following key terms: “soybeans”, “pulses”, “chickpeas”, “lentils”, “beans”, “peas”, “legumes”, “phaseolus vulgaris”, “insulin resistance”, “insulin sensitivity”, and “insulin sensitizing”. There were 8 clinical studies examining the insulin-sensitizing effects of soybeans and certain pulses (dried beans, dried peas, chickpeas), but none for lentils.

With respect to studies investigating potential mechanisms, 11 preclinical studies were identified and these studies examined only soybeans or chickpea. Upon critical evaluation of these studies, additional key terms pertinent to the identified bioactive compounds were added to the word combinations used in the literature search, such as: “isoflavones”, “anthocyanins”, “alpha-galactooligosaccharides”, and “oligosaccharides”. This secondary search yielded four additional studies that were included in this review. The 15 preclinical studies obtained were used to identify the proposed mechanism(s) of action with respect to improving insulin sensitivity, and these studies focused largely on adipose tissue and related molecules.

It is important to note that there was limited information regarding the genus, species, and variety of the legume plants investigated in these studies, thus making it difficult in some cases to determine exactly which plant was being investigated. We believe this information is necessary to properly identify the plant(s) being examined, and therefore we recommend that future publications include such information.

## 3. The Bioactive Compounds of Soybeans and Pulses Discussed in This Review

### 3.1. Isoflavones

Isoflavones are bioactive compounds that belong to a class of secondary metabolites known as flavonoids, a diverse range of polyphenolic compounds found in plants [30]. Isoflavones are present in more than 300 different types of plants [31], with legumes, such as soybeans and chickpeas, being major sources [31,32,33].

**Figure 1:**
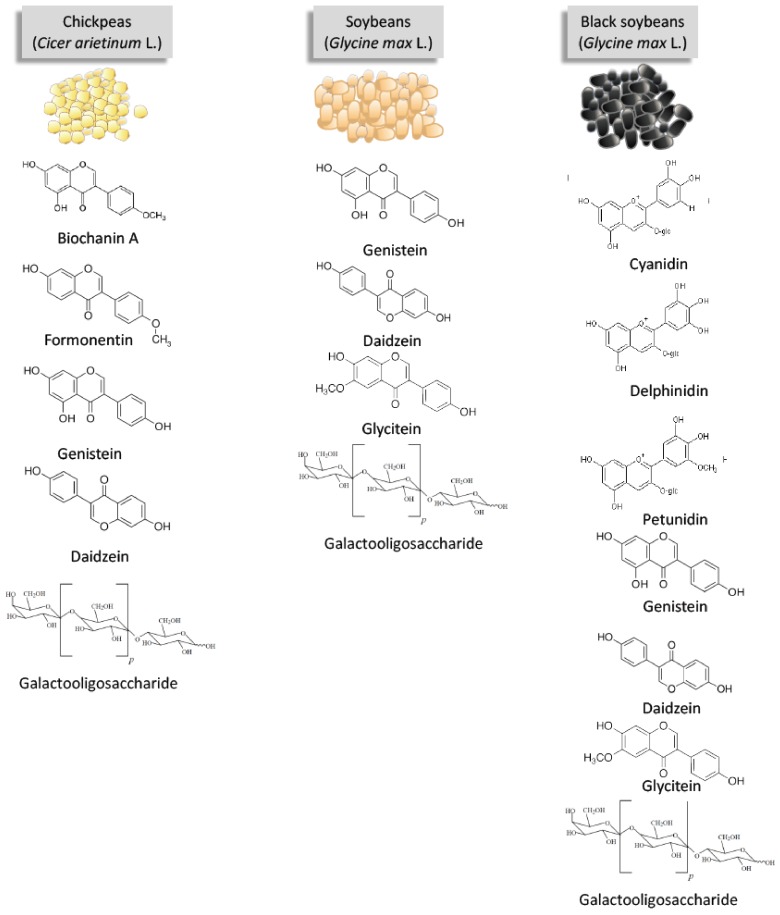
The bioactive compounds present in soybeans and chickpeas as discussed in this review.

Soybeans are the best-known source of isoflavones, including the major isoflavone aglycones, genistein, daidzein and glycitein, and their respective glycoside conjugates, genistin, daidzin, and glycitin [31,34]. Chickpeas also contain genistein and daidzein; however, the major isoflavones found in chickpeas are biochanin A (aglycone and glucoside forms) and formonentin [32]. The relevant isoflavone compounds in soybeans and chickpeas with regard to this review are summarized in Figure 1.

Isoflavones are most notable for their phytoestrogen activity, which is due to their weak affinity for estrogen receptors [33,35]. Thus, isoflavones have been commonly characterized in relation to cancers, cardiovascular disease, inflammation, and diabetes [33,35]. Their proposed anti-diabetic activity is reportedly due to their ability to attenuate insulin resistance and improve insulin secretion [32], with evidence to support these claims found in various clinical trials [5,17,18,19].

### 3.2. Anthocyanins

Anthocyanins are the most vibrant class of flavonoids, being responsible for the red, blue, and purple pigments of plants [30]. The most common sources of anthocyanins are blue-, red-, and purple-hued fruits (e.g., blueberries, blackberries, strawberries) [30,36], but they are also found in legumes, such as beans with darker-coloured seed coats (e.g., black soybeans, black beans and red kidney beans) [37,38].

The most common anthocyanins found in plants are cyanidin, delphinidin, pelargonidin, peonidin, petunidin, and malvidin [36]. The types of anthocyanins present in black soybeans remain to be elucidated; however, studies have consistently confirmed the presence of at least three anthocyanins: cyanidin-3-glucoside, delphinidin-3-glucoside, and petunidin-3-glucoside (Figure 1) [39,40].

Similar to isoflavones, anthocyanins are associated with health-promoting properties with respect to cardiovascular disease, cancer, inflammation, and diabetes, and typically ascribed to their antioxidant activity [39]. Dietary anthocyanins have also been associated with improvements in insulin sensitivity [39], making them a potential dietary ammunition against insulin resistance.

### 3.3. Galactooligosaccharides

Galactooligosaccharide (GOS) is a term that collectively describes a heterogeneous group of carbohydrates composed of 1 to 10 galactosyl moieties [41,42]. GOS are naturally found in human and bovine milk sources [42,43], but can also be found in foods such as chickpeas and soybeans [19,40], and are commercially manufactured from lactose using β-galactosidase [42]. The general structure of galactooligosaccharides can be viewed in Figure 1.

GOS function as one of the most potent prebiotics and have several health benefits including promotion of gastrointestinal health and weight management, and prevention of carcinogenesis, to name a few [44]. The ability of GOS to improve insulin sensitivity has been proposed and will be discussed within the scope of this review.

## 4. The Proposed Mechanisms of Action for Reduction of Insulin Resistance by Soybeans and Pulses

### 4.1. Glucose Transporter-4 and Glucose Utilization

Under normal conditions, pancreatic β-cells release insulin in amounts that correspond to changes in plasma glucose concentrations [45]. Glucose is then removed from the circulation by uptake into the cells of insulin-sensitive tissues (skeletal muscle, adipose) via glucose transporter-4 (GLUT4) for energy utilization or storage [46]. The presence of circulating insulin stimulates around 50% of intracellular GLUT4 to be redistributed from storage vesicles in the cytosol to the plasma membrane [46,47]. Studies have shown that in patients with type 2 diabetes, where insulin resistance is present, the expression and translocation of GLUT4 is significantly reduced [32,46]. Therefore, increasing the levels and translocation of GLUT4 is a crucial factor in regulating glucose tolerance and insulin sensitivity to prevent the development of insulin resistance [46,47,48].

Bioactive compounds from soybeans and pulses have been investigated for their efficacy in improving glucose utilization and insulin sensitivity via increasing GLUT4 gene expression and/or plasma membrane levels. Huang et al. [49] used black soybean koji (a fermented black soybean product) to prepare an extract for treating 3T3-L1 preadipocytes. Preadipocytes are commonly used in insulin resistance studies to determine effects on adipogenesis and related processes, such as glucose uptake [50,51]. Insulin resistance was induced in the preadipocytes by treating with dexamethasone and insulin for 60 h (eight days is required to obtain mature adipocytes under the culture conditions), a method shown to promote insulin resistance in this cell line [52]. The black soybean koji extract increased protein levels of GLUT4 in a dose-dependent manner from 25 to 200 μg/mL compared with the vehicle treatment in the preadipocytes [49]. Furthermore, the highest concentration of black soybean koji extract (200 μg/mL) significantly increased glucose utilization in the preadipoctyes compared with the vehicle treatment. Huang et al. [49] analyzed the black soybean extract for isoflavone content but not for other flavonoids, such as anythocyanins, which are responsible for the black seed coat colour of this soybean variety and have been shown in other studies to increase GLUT4 expression [38]. A study conducted by Inaguma et al. [53] is in accordance with the supposition that it is the anthocyanins in black soybeans that are responsible for increased glucose uptake. They treated adipocytes (3T3-L1 cells) with the anthocyanin, cyanidin 3-glucoside (20 and 100 μM), after extraction from black soybeans [53]. Both cyanidin 3-glucoside concentrations significantly increased mRNA levels of GLUT4 compared to the vehicle treatment.

Improved glucose utilization due to black soybean anthocyanins was also demonstrated in a diabetic model in vivo [38]. Diabetes was induced in male Sprague Dawley rats by injecting streptozotocin (50 mg/kg). In the diabetic control groups, blood glucose levels increased, blood insulin concentrations decreased, and cardiac and skeletal muscle GLUT4 protein levels declined [38]. Administration by oral gavage of anthocyanins (50 mg/kg) extracted from black soybeans to the diabetic rats for 30 days reduced blood glucose levels, increased circulating insulin concentrations, and increased GLUT4 translocation and insulin receptor phosphorylation in heart and skeletal muscle, compared to the diabetic control group [38].

A study using anthocyanins extracted from a non-legume source supports anthocyanins being the class of compounds responsible for improving GLUT4 levels [54]. An anthocyanin-rich purple corn water extract significantly increased glucose uptake in insulin resistant adipocytes compared to vehicle treatment [48]. Cyanidin 3-glucoside, the major anthocyanin present in the purple corn extract, administered on its own also increased glucose uptake in insulin resistance adipocytes [54].

In contrast, Gao et al. [32] reported that treatment of 3T3-L1 adipocyte cells with isoflavones extracted from chickpeas (concentrations of 50 μg/mL and 100 μg/mL) significantly reduced GLUT4 mRNA and protein levels in a dose-dependent manner compared to vehicle treated adipocytes. While it was observed that chickpea isoflavones decrease GLUT4 levels [32], the adipocytes were not made insulin resistant as was the case for the anthocyanin studies described above [38,49,53,54].

Overall, it is apparent from the above studies [32,38,49,53,54] that anthocyanins are the compound responsible for increasing the translocation and expression of GLUT4 in adipocytes under conditions of insulin resistance.

### 4.2. Peroxisome Proliferator-Activated Receptor-γ

Peroxisome proliferator-activated receptor (PPAR) is a member of the nuclear receptor family of ligand-activated transcription factors [55]. Of the three different isoforms of PPARs, PPAR-α and PPAR-γ are commonly studied for their relationships with insulin sensitivity [55,56]. PPAR-γ is predominantly found in adipose tissue and is an essential regulator of adipocyte differentiation and, thus, is an indirect regulator of glucose and lipid homeostasis [57,58]. Upon activation, PPAR-γ facilitates normal insulin sensitivity by directly modulating the activation of specific insulin signaling molecules [32,57,58].

It has been suggested that isoflavones can bind to and activate PPAR-γ [59]. In the study conducted by Huang et al. [49], the black soybean koji extract (200 μg/mL), rich in isoflavones, significantly decreased PPAR-γ protein levels in insulin resistant 3T3-L1 preadipocytes, compared with vehicle treatment. Similarly, Gao et al. [32] saw that with chickpea isoflavones (50 and 100 μg/mL) PPAR-γ mRNA and protein levels were decreased in 3T3-L1 preadipocytes, compared to control. Gao et al. [32] also showed mRNA and protein levels of CCAT-enhancer-binding protein-α (C/EBP-α; a transcription factor that controls PPAR-γ expression) were reduced by chickpea isoflavones. Separately, anthocyanins extracted from black soybeans (50 μg/mL) also decreased PPAR-γ protein levels in differentiated 3T3-L1 adipocytes [60]. The anthocyanin extract from these black soybeans included cyanidin-3-glucoside (68.3%), delphinidin-3-glucoside (25.2%), and petunidin-3-glucoside (6.5%) [60].

The studies by Gao et al. [32] and Huang et al. [49] indicated that insulin sensitivity was improved in each instance, which presents a couple of questions: (1) is adipocyte maturity a factor in insulin sensitivity; (2) would anthocyanins also inhibit adipocyte differentiation by down-regulating PPAR-γ in preadipocytes, or would isoflavones continue to down-regulate PPAR-γ in mature adipocytes?

Thiazolidinediones (TZDs) are insulin-sensitizing drugs that bind and activate PPAR-γ and stimulate adipocyte differentiation, resulting in increased accumulation of fat deposits [61,62]. Interestingly, Kadowaki et al. [59] proposed that TZDs act by increasing the number of small adipocytes via PPAR-γ, while also decreasing the number of large adipocytes. Both actions will contribute towards alleviating insulin resistance [61]. In this context, it could be speculated that the down-regulation of PPAR-γ by isoflavones from chickpeas and soybeans in preadipocytes may prevent the development of large, dysfunctional adipocytes commonly associated with obesity and insulin resistance [63]. Therefore, by inhibiting lipid accumulation, isoflavones are improving glucose utilization and insulin sensitivity [49].

It is important to note that neither study mentioned whether PPAR-γ was activated or if other insulin signaling genes were activated, and knowing these points would help elucidate the mechanism of action for how isoflavones improve insulin sensitivity via PPAR-γ.

### 4.3. Fat Deposition & Metabolism

With chronic excess energy consumption, such as that associated with obesity, triglycerides and other lipid metabolites spill over into non-adipose tissues such as the liver and muscle [55]. This ectopic lipid deposition significantly interferes with intracellular insulin signaling in these tissues, leading to insulin resistance [55].

Pre-clinical studies have demonstrated the ability of legumes and bioactive isoflavone compounds to reduce fat deposition [32,64,65,66]. Yang et al. [66] investigated the effect of a high-fat diet supplemented with raw, crushed chickpea seeds in 8-week old male Sprague Dawley rats. After 8 months of dietary supplementation, rats fed chickpeas had significantly reduced body weight, epididymal fat pad weights (an indicator of visceral adiposity), and decreased levels of triglycerides in the liver and muscle compared to non-supplemented rats fed a high-fat diet [66]. Additionally, postprandial plasma glucose and insulin levels in chickpea-supplemented rats were lower compared to non-supplemented rats [66]. These results demonstrate that chickpeas can blunt the hyperglycaemic and hyperinsulinaemic effects of a long-term high-fat diet, as well as reduce visceral adiposity and ectopic lipid accumulation [66]. An interesting observation from this study conducted by Yang et al. [66] relates to its use of raw chickpea seeds. While some countries do consume raw chickpea seeds, it is typically advised to cook chickpeas and other pulses to inactivate anti-nutritional factors that can cause undesirable gastrointestinal effects if consumed [67,68]. It is unclear in this study whether the observed effects are due to the chickpeas or the anti-nutritional compounds. Separately, Gao et al. [32] showed that treatment of 3T3-L1 adipocytes with isoflavones extracted from chickpeas (extract concentrations of 50 μg/mL and 100 μg/mL), intracellular lipid accumulation was reduced in a dose-dependent manner compared to control adipocytes.

Soy isoflavones have also been investigated for their in vivo effects on fat deposition and subsequent insulin resistance. In high-fat diet-induced insulin resistant male Sprague Dawley rats, administration of soybean isoflavones (150 mg/kg and 450 mg/kg) by oral gavage for 30 days significantly reduced total white adipose tissue weight, including epididymal and peri-renal fat pad weights, compared to the insulin resistant control group, with no difference in body weights among the groups [1]. Additionally, fasting insulin levels and HOMA-IR were significantly lower in the rats receiving soybean isoflavones compared to their insulin resistant control counterparts [1]. The effect of soy isoflavones was also investigated in obese and lean spontaneously hypertensive/NIH-corpulent (SHR/N-cp) rats [64]. Dietary soy isoflavone supplementation in both the lean and obese SHR/N-cp rats significantly reduced fat deposition in several fat depots compared to control rats [64]. The soy isoflavone mixture consisted of genistein, daidzein, and glycitein, and was administered at 0.1% *w*/*w* (100 mg isoflavones per kg of diet) in AIN-93G semi-purified diet [64]. This amount equates to around 2.5 mg isoflavones/day based on an approximate average intake of 25 g diet/day [69]. While there were no data to confirm the presence of insulin resistance in the obese rats, these data in obese SHR/N-cp rats are of interest as this is an insulin-resistant phenotype and the results imply soy isoflavones are efficacious in reducing adiposity in an insulin-resistant state [64].

As previously mentioned, genistein is one of the main isoflavone glucoside conjugates found in soybeans and, to a lesser extent, in chickpeas [1,32,64]. Using genistein (90% pure) as a dietary supplement (0.1% *w*/*w* in high-fat diet) for 4 weeks, Choi et al. [65] found that female ovariectomized Sprague Dawley rats had significantly smaller adipocytes, but not fat pad mass, compared to their ovariectomized control counterparts. Smaller adipocytes are more insulin sensitive, thus, it is not surprising that HOMA-IR index was significantly decreased in ovariectomized rats supplemented with genistein, reaching a level comparable with the non-ovariectomized sham group fed the high-fat diet [65]. As well, the addition of genistein to the high-fat diet resulted in positive changes to levels of enzymes related to fat synthesis and oxidation, such as reduced hepatic fatty acid synthase activity and increased carnitine palmitoyltransferase, β-oxidation, and succinate dehydrogenase activity in adipose. Genistein supplementation also resulted in down-regulation of genes responsible for fatty acid synthesis, and up-regulation of genes responsible for fat utilization [65]. While this study was not conducted with genistein extracted from soybeans or chickpeas, it does help elucidate the potential mechanism by which genistein affects fat metabolism, with subsequent improvements in insulin resistance.

Not all studies have reported a positive effect of soy and soy-derived isoflavones on fat deposition. Zanella et al. [70] showed that male C57BL/6 J mice fed a low-fat diet containing soybeans (8.5% *w*/*w*) for 21 weeks had significantly increased total fat mass and fat pad weights, but not lean mass or total body weight, compared to mice fed the soy-free diet. A similar result was seen when additional genistein was supplemented (5 mg/kg/day) by oral gavage. Neither the soy nor genistein treatments influenced glucose metabolism or insulin sensitivity, as determined by postprandial glucose and insulin tolerance testing, respectively [70].

The collective findings from the above soy studies [58,59,64] present the possibility that with respect to adiposity there is no benefit to providing soy in a non-diseased animal model consuming a low-fat diet [70]. However, the evidence supports the conclusion that under conditions of a high-fat diet, supplementing with soy, chickpeas, and/or their respective isoflavones can attenuate insulin resistance, possibly by reducing adiposity [32,64,65,66].

### 4.4. Adipokines

The relationship between adipose tissue and insulin resistance is well-known: the verdict being that with increased adipose mass, and thus weight gain, there is an impairment of insulin action, leading to insulin resistance [71]. However, increased mass is just one piece of the puzzle in understanding the role adipose plays with insulin resistance. Adipose tissue is an active endocrine organ, producing and secreting proteins known as adipokines [72,73]. Adipocytes become hypertrophic and dysfunctional as adiposity increases, leading to dysregulation of adipokines [74,75]. Therefore, in the presence of increased adiposity and insulin resistance, certain adipokines are affected, including adiponectin, leptin, and resistin [72,75]. Given the connection between diet, adiposity, and insulin resistance, it is not surprising that dietary components can influence adipokine levels [76], and thus, have a role in insulin resistance.

#### 4.4.1. Adiponectin

Adiponectin is recognized for its many beneficial biological effects, including anti-inflammatory, anti-atherogenic, and anti-diabetic actions [77]. Levels of adiponectin, as measured in the circulation and in adipose tissue, are inversely related to insulin resistance [49,59]. Thus, restoring adiponectin levels is beneficial for attenuating insulin resistance and improving insulin sensitivity [78].

In high-fat diet-induced insulin resistant Sprague Dawley rats, the administration of soybean isoflavones (150 mg/kg/day and 450 mg/kg/day) by oral gavage for 30 days increased both circulating protein and mRNA levels of adiponectin in peri-renal white adipose tissue compared to the insulin resistant rats administered vehicle [1]. Additionally, the soy isoflavones reduced HOMA-IR compared to the insulin resistant control group and there was a significant negative correlation between circulating adiponectin levels and HOMA-IR [1].

The effect of soybean extract was also determined in insulin resistant 3T3-L1 adipocytes in vitro [49,53]. Treatment for 60 h with isoflavone-rich black soybean koji extract (50 to 200 μg/mL) significantly increased adiponectin protein levels compared to the vehicle treatment in the study conducted by Huang et al. [49]. In the study by Inaguma et al. [53], the authors referenced a separate study by Han et al. [79] where cyanidin 3-glucoside, the anthocyanin extracted from black soybeans, significantly increased adiponectin mRNA levels in 3T3-L1 cells in a dose-dependent manner; unfortunately, we were unable to obtain a copy of this article and therefore cannot comment further on the details of the study.

Based on the above studies, it is not possible to determine which compound(s) present in soy (i.e., anthocyanins or isoflavones), is responsible for the observed increases in adiponectin levels. However, not all studies reported a positive effect of soy isoflavones on adiponectin levels. Using genistein (90% pure) as a dietary supplement (0.1% *w*/*w* in high-fat diet) provided to ovariectomized Sprague Dawley rats for 4 weeks, Choi et al. [65] found no significant differences in the levels of serum adiponectin between control and genistein-supplemented groups. There was, however, an improvement in the insulin resistance index in ovariectomized rats supplemented with genistein [65]. Kavanagh et al. [80] also noted a similar result with regards to adiponectin in mature, premenopausal, insulin-resistant female monkeys supplemented with dietary soy isoflavones (155 mg/day) for 4 months. The dietary supplementation had no effect on plasma adiponectin levels; it did, however, increase insulin area under the curve compared to control group, while there were no differences in glucose area under the curve [80]. The findings from Kavanagh et al. [80] suggest that soybean isoflavones in this instance promote insulin hypersecretion in premenopausal female monkeys.

Tissue levels of adiponectin were not examined in the studies conducted by Choi et al. [65] and Kavanagh et al. [80]. Studies have reported that adiponectin circulates until it binds to specific cell-surface receptors [77]. These adiponectin receptors have been identified in insulin-responsive tissues such as liver, adipose, and skeletal muscle [77]. Therefore, the improvement in the insulin resistance index reported by Choi et al. [65] might be explained by increased adiponectin responsiveness in these tissues.

#### 4.4.2. Leptin

Leptin is commonly known for its effects on energy balance and the subsequent relationship with weight gain and adiposity [75,81]. Evidence surrounding the relationship between leptin and insulin resistance is controversial, but it is thought that plasma leptin concentrations are positively correlated with insulin resistance [59,82].

Zhang et al. [1], as previously discussed with adiponectin, measured leptin levels in high-fat diet-induced insulin resistant rats. It was shown that a high dose of soy isoflavones (450 mg/kg/day) for 30 days increased circulating protein and adipose mRNA levels of leptin, in spite of reduced adipose weight, compared to the insulin resistant control group [1]. While HOMA-IR levels were reduced with medium and high doses of soy isoflavones (150 and 450 mg/kg/day, respectively), the authors reported that the negative correlation observed between circulating leptin and HOMA-IR failed to reach statistical significance (*p* = 0.053) [1]. Serum leptin levels were also measured following the dietary supplementation of genistein in ovariectomized rats fed a high-fat diet [65]. Choi et al. [65] noted there were no differences in serum leptin levels between groups after 4 weeks of supplementation. In a separate study conducted by Yang et al. [66], 8-week old male Sprague Dawley rats fed a high-fat diet supplemented with raw, crushed chickpea seeds (10% *w*/*w*) for 8 months had lower leptin mRNA levels in adipose compared to the untreated high-fat diet group. Chickpea supplementation also resulted in lower HOMA-IR levels, indicating improved insulin sensitivity [66].

The above studies show a discrepancy regarding the potential relationship between leptin and insulin resistance, and the effects of chickpea and soybean isoflavones on leptin levels [1,65,66]. Zhang et al. [1] indicated that there is a trend, though not statistically significant (*p* = 0.053), between increased leptin levels and improved insulin sensitivity, which is similar to observations by other authors showing leptin improves insulin sensitivity [75,82]. While Zhang et al. [1] saw changes in leptin levels with soybean isoflavones, Choi et al. [65] reported genistein improved insulin sensitivity but had no effect on leptin levels. Yang et al. [66] showed reduced leptin and HOMA-IR levels from chickpea isoflavones but no correlation analyses were conducted; thus, evidence is inconclusive regarding the impact of leptin on insulin resistance. Without a confirmed relationship between leptin and insulin resistance, there is not enough evidence to determine if one particular isoflavone source is more beneficial than the other.

#### 4.4.3. Resistin

Resistin is a lesser known adipokine that has been observed to promote insulin resistance [75]. Unfortunately, there are limited studies surrounding the effects of soybeans and pulses on resistin levels. In the study conducted by Zhang et al. [1], both concentrations of soy isoflavones (150 mg/kg/day and 450 mg/kg/day) significantly lowered plasma resistin levels after 30 days of treatment. Meanwhile, only the higher dose (450 mg/kg/day) of soy isoflavones lowered adipose mRNA levels of resistin [1]. Additionally, there was a positive correlation between plasma resistin levels and HOMA-IR, suggesting that increased resistin secretion promotes insulin resistance [1].

Overall, the collective findings from the above adipokine studies indicate that isoflavone compounds from soy have favourable effects on insulin sensitivity, likely by up-regulating adiponectin and down-regulating resistin [1,49,53]. However, the evidence surrounding the effects of chickpea and soybean isoflavones on leptin levels, and the association with insulin resistance, is inconclusive at this time and requires further investigation.

### 4.5. Short-Chain Fatty Acids and Gut Microflora

Gut microflora have a substantial impact on health, with the nature of their effects determined by the specific bacteria colonizing in the gut [83]. The diet has a strong impact on the bacterial environment of the gut [83], for example, by affecting the production of short-chain fatty acids (SCFA) by intestinal fermentation [83,84]. SCFA (acetic, propionic, butyric acids) provide energy to colonic cells and acidify the luminal pH, thereby suppressing the growth of pathogenic bacteria and promoting the growth of beneficial bacteria [41,83]. Evidence suggests that a high-fat diet shifts the composition of gut microflora from fewer beneficial bacteria (e.g., Bifidobacteria, Lactobacillus, Bacteroidetes) to more of the harmful bacteria (e.g., Firmicutes, Clostridium) [41,84]. This dysbiosis is a key factor in the development of insulin resistance and other metabolic diseases [41,85].

GOS are thought to act as prebiotics by enhancing the production of SCFA and thus favouring the growth of beneficial bacteria [41,84]. Zhou et al. [86] investigated the effects of commercially available soybean galactooligosaccharides (SBOS) on the gut ecosystem of Huangjiang mini-piglets (an experimental model representative of human intestinal physiology [87]). The piglets were fed a standard diet and randomly assigned to supplementation of corn starch (0.5% *w*/*w*; control group) or SBOS (0.5% *w*/*w*; experimental group) for 14 days [86]. The authors reported SBOS supplementation increased total SCFA, propionate, and butyrate concentrations in the ileum and colon, as well as acetate and valerate concentrations in the ileum compared to the control group. SBOS supplementation also increased numbers of beneficial intestinal bacteria including *Bifidobacterium*, *Faecalibacterium*, *Fusobacterium*, and *Roseburia*, while decreasing numbers of potentially harmful bacteria such as *Clostridium*, *Streptococcus*, and *E. coli* compared to the control group [86].

A similar effect was observed in the study conducted by Dai et al. [41], where they investigated the effect of alpha-GOS (α-GOS) extracted from dried chickpea powder in CD-1 IGS mice fed a high-fat diet for 6 weeks. The CD-1 IGS mouse is an outbred, general, multi-purpose model that presents as a healthy phenotype [88]. As expected, consumption of a high-fat diet for 6 weeks reduced SCFA and decreased total bacterial quantity and altered gut microbiota composition [41]. Supplementation with chickpea α-GOS (0.083 g/kg/day to 0.83 g/kg/day) concurrent with HFD feeding for 6 weeks promoted the secretion of SCFA in a dose-dependent manner compared to the high-fat diet and normal chow groups. All chickpea α-GOS treatments also significantly stimulated the growth of beneficial bacteria, Bifidobacterium and Lactobacillus, compared to the high-fat group. Although the HOMA-IR was elevated in the high-fat diet group compared to the normal control group, all the chickpea α-GOS groups had HOMA-IR values intermediate between the high-fat diet group and the normal control group, but this was not considered statistically significant [41]. A similar study using male C57BL/6J mice observed that a longer duration, 18 weeks compared to 6 weeks, of high-fat feeding was required to see statistically significant effects on insulin parameters from dietary GOS supplementation (7% *w*/*w*) [89]. Additionally, the supplemented dose of GOS was higher in the study conducted by Kavadi et al. [89], approximately 210–350 mg/day (based on an average feed intake of 3–5 g/day [90]) compared to around 21 mg/day for the study by Dai et al. [41]. Thus, it is possible that with a longer study duration and/or higher dose of GOS the chickpea α-GOS treatments could have reached statistical significance for HOMA-IR. Dai et al. [41] also reported no significant differences in body weight among the high-fat diet and three α-GOS treatment groups. The lack of statistical effect on HOMA-IR by chickpea α-GOS may be due to the weak reduction in body weight, or possibly adiposity; however, adiposity was not addressed in the study.

While a possible link between consumption of GOS from soybeans and chickpeas and improved insulin resistance was not examined in the studies by Zhou et al. [86] and Dai et al. [41], respectively, other studies have investigated the mechanism by which SCFA improve insulin resistance. Over the course of 20 weeks, supplementing a high-fat diet with 5% *w*/*w* SCFA (acetate and propionate) in male C57BL/6J mice reduced HOMA-IR levels to that comparable with mice fed a low-fat diet, indicating improved insulin sensitivity [84]. Further investigation into the mechanism of action revealed that SCFA supplementation not only reduced total adipocyte numbers but also promoted smaller adipocytes rather than the abundance of large adipocytes observed in mice fed a supplement-free high-fat diet [84]. There was also evidence of SCFA promoting adipocyte browning, with increased cytochrome c oxidase activity (an indicator of mitochondrial respiratory capacity) and expression of browning markers (e.g., *Pgc1α*). Lastly, it has been proposed that propionate plays a crucial role in the mechanism underlying the prevention of insulin resistance by SCFA, as evidenced by increased levels of hepatic odd-chain fatty acids (a biomarker for propionate formation) and a negative correlation between the formation of those odd-chain fatty acids in the liver and the secretion of insulin during oral glucose tolerance testing [84].

In contrast, studies have also demonstrated that GOS from sources other than soybeans and chickpeas have no effect on improving insulin sensitivity. In a study conducted by Stahel et al. [91], Sprague Dawley rats were fed a diet supplemented with GOS (15% *w*/*w*) for nine weeks. GOS improved the microbial gut profile by increasing the abundance of *Bifidobacterium* spp. *Lactobacillus* spp., and Bacteroidetes, but it had no effect on insulin sensitivity, compared to the control group (supplemented with 15% *w*/*w* methylcellulose) [91]. As seen with the study by Dai et al. [41], the study length may not be optimal for seeing a change in insulin sensitivity in a population without insulin resistance. However, a similar finding was observed in overweight or obese prediabetic men who received GOS supplements (15 g/day) with their regular meals for 12 weeks [92]. Supplementing with GOS increased fecal *Bifidobacterium* spp., but there were no changes in insulin sensitivity [92]. It is possible that GOS from legumes, rather than from milk sources, may be more beneficial for improving insulin sensitivity; variability in the properties (structural, functional) of GOS from different sources may play a role in their efficacy. Further research is required to confirm the effect of SBOS and chickpea α-GOS on insulin sensitivity, as well as the required conditions for SBOS and chickpea α-GOS to have an optimal effect.

## 5. General Summary and Conclusions

**Figure 2:**
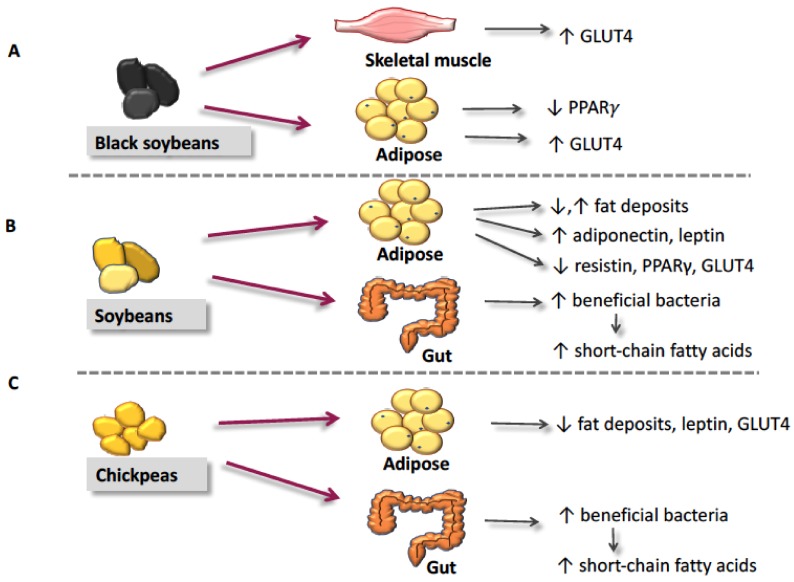
The proposed processes initiated by soybeans and chickpeas that lead to improved insulin sensitivity. (**A**) The actions of black soybeans in target tissues. (**B**) The actions of yellow soybeans in target tissues. (**C**) The actions of chickpeas in target tissues. Abbreviations: ↓, decrease; ↑, increase; GLUT4, glucose transporter-4; PPARγ, peroxisome proliferator-activated receptor-γ.

Soybeans and pulses have proven to be effective at improving insulin sensitivity, clinically evidenced by reductions in HOMA-IR (Table 1) [5,17,18,19,20,23,25,26]. Preclinical studies have attributed the insulin-sensitizing properties of soybeans and pulses, particularly chickpeas, to their bioactive compounds [1,32,38,41,49,53,60,64,86]. This review has summarized the current literature surrounding soybeans, chickpeas, and their bioactive compounds to improve insulin sensitivity (Table 2 and Table 3). The proposed insulin-sensitizing actions of these leguminous plants are summarized in Figure 2 and Table 4.

In general, soybeans and chickpeas demonstrated efficacy on pathways related to improved insulin sensitivity when a disease condition was present [1,38,41,49,64,66]. In models presenting a disease condition, soybeans, chickpeas, and/or their bioactive compounds, could reduce adiposity [1,32,62,64], positively affect adipokines [1,49], inhibit adipogenesis (via down-regulation of PPAR-γ) [32,60], increase GLUT4 levels [38,49,53], and increase SCFA-producing beneficial bacteria in the gut [41]. In models where a disease condition was not present there were contrary effects from soybean and chickpea supplementation such as increased adiposity [70] and decreased leptin levels [66]. In vitro studies that did not specify the application of an induced disease state were considered to be absent of a disease condition [32,53,60]. From the observed findings, it is possible that in the absence of an underlying disease condition, consuming soy, chickpeas, and/or their bioactive compounds does not impose a functional benefit on insulin sensitivity, aside from providing the general nutritive value of the legumes. 

Nowadays, many studies infer the actions of soybeans and pulses are due to the high antioxidant activity of their bioactive compounds, such as the anthocyanins and isoflavones [13,93,94]. While these compounds may attenuate the oxidative stress that is associated with insulin resistance [95], the bioactive compounds of soybeans and pulses have biological effects beyond antioxidant activity and the contribution of these mechanisms inwards improving insulin sensitivity should be acknowledged. As previously discussed, isoflavones from soybeans and chickpeas reduced adiposity under disease conditions [1,32,64,66], an action beneficial for improving insulin resistance. Isoflavones are suggested to inhibit lipid accumulation in adipocytes through inhibiting PPARγ (a marker of early- and mid-stage differentiation) [49,62], glycerol-3-phosphate dehydrogenase (a marker of late-stage differentiation) [88], and inducing apoptosis of mature adipocytes [96]. It has been demonstrated that isoflavones regulate PPARγ by inhibiting tyrosine phosphorylation of C/EBPβ, and activating Wnt signaling and adenosine monophosphate-activated protein kinase (AMPK) pathways [97,98]. These actions produce anti-adipogenic effects that improve insulin resistance. The activation of AMPK may also be a key mechanism of action for anthocyanins. Studies have shown AMPK is activated by anthocyanins [99,100], which then increases GLUT4 translocation leading to increased glucose uptake and improved insulin sensitivity [99,101]. It is also suggested that anthocyanins may indirectly activate AMPK by increasing adiponectin secretion [100].

While the summarized in vitro and in vivo evidence (Table 2 and Table 3) supports the potential insulin-sensitizing effects of both chickpeas [32,41,66] and soybeans [1,38,49,53,60,64], soybeans appear to have a more robust effect on insulin sensitivity. These findings may be due to the larger number of research studies available for soybeans compared to chickpeas; however, they may also be due to the presence of additional bioactive compounds, such as anthocyanins [38,53,60], within certain varieties of soybeans (e.g., black soybeans) that are otherwise not present in chickpeas.

The question of the human relevance of the bioactive compounds used in the in vivo studies is important to consider with respect to implementing these results within the context of human consumption. Various studies have reported the mean dietary intake of isoflavones, mostly from soy sources, to be less than 5 mg/day in the United States and across European populations [102,103,104,105]. In Asian populations, the mean isoflavone intake from dietary sources ranges from 22–47 mg/day [104,105], and among vegetarian-based diets the mean dietary intake is around 22 mg/day [105]. Interestingly, isoflavone intakes average around 50 mg/day among supplement users in Western countries, with certain supplements containing up to 107 mg of isoflavones [105]. In the various studies that examined isoflavones in vivo, the highest supplement dose that produced an effect was 450 mg/kg body weight of soy isoflavones in Sprague Dawley rats (150–180 g body weight) [1]. This dose is equivalent to approximately 68–81 mg of isoflavones/day, similar to the isoflavone supplements provided to participants in the clinical studies listed in Table 1 [5,18,20]. As for anthocyanins, average dietary intakes have been reported around 12 mg/day in the United States [106] and 30 mg/day in European countries [107]. There are no recommendations in place for Canada, the United States, or the European Union regarding anthocyanin intake; however, in China, the dietary recommendation is 50 mg anthocyanins/day [106]. Human and animal studies have reported no toxic effects from high doses of anthocyanins (9 g/kg for rodents and around 2 g/day for humans) [106,108]. Foods such as blueberries, blackberries, and black soybeans contain on average (per 100 g) 353 mg, 529 mg, and 23 mg of anthocyanins, respectively [109]. In the study conducted by Nizamutdinova et al. [38], animals consumed 50 mg/kg/day of anthocyanins from black soybeans, equating to approximately 11–12.5 mg anthocyanins/day. In terms of oligosaccharide dietary intakes, little information is available; however, around 3 g/day is suggested in the European diet for healthy gut microflora [110]. In the study by Dai et al. [41], mice consumed 0.083–0.83 g/kg α-GOS from chickpeas per day, equating to approximately 2.1–21.0 mg/day. Han & Baik [111] observed 144.9 mg/g of total oligosaccharides in dry chickpeas; therefore, one cup (250 g) of dry chickpeas would have approximately 36 g of oligosaccharides. However, it is important to note that oligosaccharide content does decrease substantially with cooking [111]. Overall, the dosages of isoflavones, anthocyanins, and α-GOS from the in vivo studies discussed in this review are relevant to the intakes obtainable by humans, either by dietary sources or supplements.

It is known that cardiovascular disease is the prevalent cause of morbidity and mortality for individuals with diabetes [112]. Studies have shown that consumption of soybeans and the four listed pulses can improve vascular function [113,114,115,116]. Therefore, it is noteworthy to mention that while soybeans and chickpeas have proven beneficial for insulin sensitivity, soybeans and the four pulses (lentils, dried beans, dried peas, chickpeas) have positive vascular effects that may attenuate the cardiovascular complications associated with insulin resistance and diabetes.

In conclusion, the above studies have greatly increased our understanding of the role of soybeans and chickpeas for attenuating insulin resistance, and have provided convincing evidence that these leguminous plants have value as a nutritional approach for restoring insulin sensitivity that may provide even greater benefits when considering their ability to improve certain complications caused by insulin resistance. At the same time, similar information regarding the effect of the other pulses is lacking. Consequently, additional intervention studies are needed to evaluate the clinical relevance of soybeans and pulses as a means of improving insulin sensitivity and to better define the relative importance of the underlying mechanisms and pathways responsible for modulating the cellular response to insulin.

## Figures and Tables

**Table 1:** Clinical trials investigating the efficacy of soybeans and pulses to improve insulin sensitivity.

Legume	Author	Study Design	Participants and Disease State/Condition	Intervention	Results
Soy (*Glycine max.* L.)	Llaneza et al. [5]	⋅ Randomized (R), controlled, single-blinded (SB) ⋅ Longitudinal study (24 months)	⋅ Postmenopausal women (50–64 years old) ⋅ Normal to obese body mass index (22.5 to 43.5 kg/m^2^)	⋅ Control: physical exercise + Mediterranean diet ⋅ Intervention: control + 80 mg soy isoflavone extract/day (ISO)	⋅ ISO ↓ fat mass, fasting serum glucose & insulin, and HOMA-IR vs. baseline ⋅ ISO ↓ fat mass, and HOMA-IR vs. control at 24 months ⋅ ISO had greater ↓ effect on HOMA-IR, and fasting serum glucose & insulin in obese participants
	Choi et al. [17]	⋅ R, double-blinded (DB), placebo-controlled (PC) ⋅ 12-week study	⋅ Men and women (mean age range 43–53 years old) ⋅ Overweight (body mass index 25.0–29.9 kg/m^2^) ⋅ Mild hyperglycemia (fasting blood glucose 5.5–6.9 mmol/L)	⋅ Placebo: 2 g starch capsules/day ⋅ Positive control: 300 mg banaba extract/day (BE) ⋅ Intervention: 2 g soybean leaf extract/day (SLE)	⋅ No effect of interventions on body weight or body mass index ⋅ SLE and BE ↓ body fat, blood glucose and HOMA-IR vs. placebo at 12 weeks
	Choquette et al. [18]	⋅ R, DB, PC ⋅ 6-month study	⋅ Postmenopausal women (50–70 years old) ⋅ Overweight and obese (body mass index 28–40 kg/m^2^)	⋅ Controls: Placebo capsules; Exercise (resistance and aerobic training 3×/week) + placebo capsules ⋅ Interventions: 70 mg soy isoflavones/day (ISO); Exercise + ISO	⋅ ISO, without exercise, improved fasting plasma insulin and HOMA-IR vs. baseline
	Fei et al. [19]	⋅ R, controlled ⋅ 8-week study	⋅ Pregnant women (single fetus) ⋅ Gestational diabetes (diagnosed according to National Diabetes Data Group standard)	⋅ Control: insulin (3×/day) ⋅ Intervention: 10 g soybean oligosaccharides/day in water (SOGS) + insulin (3×/day)	⋅ SOGS + insulin ↓ fasting plasma insulin and HOMA-IR vs. control group at 8 weeks ⋅ SOGS + insulin ↓ total insulin dosage vs. control group
	Jamilian et al. [20]	⋅ R, DB, PC ⋅ 12-week study	⋅ Women (18–40 years old) ⋅ Polycystic Ovary Syndrome	⋅ Placebo: 50 mg capsules (composition not disclosed) ⋅ Intervention: 50 mg soy isoflavones/day (ISO)	⋅ ISO ↓ serum insulin and HOMA-IR vs. placebo at 12 weeks
	Ye et al. [27]	⋅ R, DB, PC ⋅ 24-week study	⋅ Pre- and post-menopausal Chinese women (30–70 years old) ⋅ Impaired glucose regulation (fasting glucose 5.6–7.0 mmol/L, 2-h postprandial glucose 7.8–11.0 mmol/L, or newly diagnosed diabetes not requiring medication)	⋅ Control: 10 g soy protein ⋅ Interventions: control + 50 mg/day daidzein; control + 50 mg/day genistein	⋅ No differences in fasting glucose or insulin levels ⋅ No differences in insulin sensitivity
Beans (*Phaseolus vulgaris* L.)	Bourdon et al. [28]	⋅ Cross-over (CO) ⋅ 3 × 6-h visits + 3 × 1–3 week washouts	⋅ Men (21–45 years old) ⋅ Healthy (body mass index 22.6–29.4 kg/m^2^)	⋅ Control: instant rice and dry milk + test meal ⋅ Intervention: 60 g white bean flakes + test meal	⋅ No differences in fasting postprandial blood glucose or insulin levels between meals
	Nilsson et al. [21]	⋅ R, CO ⋅ 2 × 3-h visits	⋅ Men and women (mean age 24 ± 1 years old) ⋅ Healthy (body mass index 22.5 ± 0.6 kg/m^2^)	⋅ Evening meals: ⋅ Control: 89 g white wheat bread (WB) ⋅ Intervention: 101 g cooked Swedish brown beans ⋅ Next-day: standardized breakfast	⋅ Brown beans ↓ postprandial glucose and insulin incremental area under the curve (0–120 min) vs. WB ⋅ No differences in fasting glucose and insulin concentrations between evening meals
	Reverri et al. [22]	⋅ R, controlled, cross-over (CO) ⋅ 3 × 5-h visits + 3 × 1-week washouts	⋅ Men and women (mean age 49 ± 14 years old) ⋅ Metabolic syndrome (body mass index 32.2 ± 5.7 kg/m^2^; insulin resistant)	⋅ Controls: fibre-matched meal (FM) and antioxidant-matched meal (AM; 300 mg grape seed extract supplemented) ⋅ Intervention: black bean meal (BB)	⋅ No difference in postprandial blood glucose levels between meals ⋅ BB ↓ plasma insulin vs. controls
Beans (*Phaseolus vulgaris* L.) and Peas (*Pisum sativum* L.)	Winham et al. [29]	⋅ R, CO, 33 × block design ⋅ 3 × 8-week arms + 2 × 2-week washouts	⋅ Men and women (22–65 years old) ⋅ Moderately insulin resistant (fasting insulin ≥ 15 µU/mL and ≤50 µU/mL)	⋅ Control: ½ cup canned sliced carrots/day ⋅ Interventions: ½ cup canned pinto beans; ½ cup canned black-eyed peas	⋅ No difference in fasting blood glucose and insulin levels ⋅ No difference in HOMA-IR
Peas (*Pisum sativum* L.)	Marinangeli & Jones [23]	⋅ R, SB, CO ⋅ 3 × 4-week arms + 3 × 4-week washouts	⋅ Men and women (mean ages 51.8 ± 12.3 and 52.3 ± 10.0 years old, respectively) ⋅ Overweight (body mass index 25–40 kg/m^2^) ⋅ Hypercholesterolemic	⋅ Control: white wheat flour muffins ⋅ Intervention: whole pea flour (WPF) or fractioned pea flour (FPF) muffins; 50 g dried yellow peas/day	⋅ No change in body weight ⋅ No differences in postprandial blood glucose levels ⋅ WPF and FPF ↓ fasting plasma insulin and HOMA-IR vs. control
Chickpeas (*Cicer arietinum* L.)	Johnson et al. [24]	⋅ R, SB, CO ⋅ 3 × 175-min visits + 3 × 7-day (minimum) washouts	⋅ Men and women (mean age 32 ± 2 years old) ⋅ Healthy (body mass index 24.7 ± 0.8 kg/m^2^)	⋅ Control: 3–4 toasted slices of white bread (WB) ⋅ Interventions: 3–4 toasted slices of chickpea bread (CB) or extruded chickpea bread (EXB)	⋅ CB and EXB ↓ incremental plasma glucose concentrations at 90 min and 120 min, respectively, vs. WB ⋅ CB ↑ serum insulin incremental area under the curve vs. WB
	Nestel et al. [25]	⋅ Acute study: 3 × 3-h visits ⋅ Long-term study: R, CO, 2 × 6-week arms	⋅ Men and women (acute study mean age 62 ± 6 years old; long-term study mean age 57 ± 8 years old) ⋅ Healthy (acute study body mass index 26.5 ± 3.8 kg/m^2^; long-term study body mass index 25.6 ± 3.2 kg/m^2^)	⋅ Acute study ⋅ Control: white bread ⋅ Interventions: cooked, mashed chickpeas (200 g) or wheat cereal with wheat bran ⋅ Long-term study ⋅ Interventions: chickpea-foods (from 140 g canned chickpeas) or wheat-based foods	⋅ Acute study: Chickpea treatment ↓ postprandial plasma glucose levels vs. wheat and control treatments at 30 and 60 min; chickpea treatment ↓ fasting plasma insulin levels and HOMA-IR vs. wheat and control treatments ⋅ Long-term study: No differences in fasting plasma glucose or insulin levels or HOMA-IR
	Pittaway et al. [26]	⋅ CO ⋅ 20-week study (4 weeks regular diet + 12 weeks intervention + 4 weeks regular diet)	⋅ Men and women (30–70 years old) ⋅ Overweight (mean body mass index 26.3 ± 4.8 kg/m^2^) ⋅ Mildly hypercholesterolemic (mean fasting serum total cholesterol 6.5 ± 1.4 mmol/L) ⋅ Normoglycemic	⋅ Control: regular diet ⋅ Intervention: average 119 g canned chickpeas/day (Chickpea diet)	⋅ Chickpea diet ↓ fasting serum insulin and HOMA-IR vs. regular diet after 12 week intervention

Abbreviations: ↓, decrease; ↑, increase; AM, antioxidant-matched; BB, black bean meal; BE, banaba extract; CB, chickpea bread; CO, cross-over; DB, double-blind; EXB, extruded chickpea bread; FM, fibre-matched; FPF, fractioned pea flour; HOMA-IR, homeostasis modelling assessment—insulin resistance; ISO, soy isoflavones; PC, placebo-controlled; R, randomized; SB, single-blind; SLE, soy leaf extract; SOGS, soybean oligosaccharides WB, white bread; WPF, whole pea flour.

**Table 2:** Summary of the in vivo studies included in the present review.

Legume Source	Reference	Study Design	Parameters Measured	Key Findings
Soybean (*Glycine max.* L.)	Zhang et al. [1]	⋅ Male Sprague Dawley rats (150–180 g) ⋅ Groups: (1) Basal control (normal diet); (2) HFD insulin-resistant (IR control; sterilized water as vehicle control); (3) HFD + low-dose ISO (50 mg/kg/day intragastric administration (i.g.)); (4) HFD + medium-dose ISO (150 mg/kg/day i.g.); (5) HFD + high-dose ISO (450 mg/kg/day i.g.) ⋅ 30-day study	⋅ Insulin resistance index (HOMA-IR) ⋅ Fasting insulin (RIA kit) ⋅ Fasting plasma glucose (commercial reagent paper) ⋅ Fasting plasma adiponectin, leptin, resistin (rat ELISA kits) ⋅ Tissue weights (epididymal and peri-renal fat pads) ⋅ mRNA levels in peri-renal adipose tissue (quantitative real-time PCR (RT-PCR); 45 cycles)	⋅ ISO ↓ fat pad weights, no differences in body weights among groups ⋅ ISO ↓ fasting insulin and HOMA-IR vs. IR control ⋅ ISO (150 and 450 mg/kg/day) ↑ plasma and mRNA adiponectin and leptin levels, ↓ plasma resistin levels ⋅ ISO (450 mg/kg/day) ↓ mRNA resistin levels ⋅ Positive correlation between plasma resistin and HOMA-IR ⋅ Negative correlation between plasma adiponectin and HOMA-IR
	Nizamutdinova et al. [38]	⋅ Male Sprague Dawley rats (220–250 g) ⋅ Streptozotocin (STZ; 50 mg/kg) injected intraperitoneally to induce diabetes ⋅ Anthocyanins extracted from black soybeans ⋅ Groups: (1) Control (no STZ); (2) Diabetes control (STZ); (3) Anthocyanin (50 mg/kg) pre-diabetes (ANT-PRE) + STZ; (4) STZ + Anthocyanin (50 mg/kg) post-diabetes (ANT-POST) ⋅ 30-day study	⋅ Protein levels (Western blotting) ⋅ Fasting serum insulin (ELISA kit) ⋅ Fasting blood glucose (glucometer)	⋅ Anthocyanins ↓ blood glucose levels of diabetic rats vs. diabetic control group ⋅ Anthocyanins ↑ fasting serum insulin of diabetic rats vs. diabetic control group ⋅ Anthocyanins ↑ GLUT4 levels in skeletal muscle vs. diabetic control group
	Ali et al. [64]	⋅ Male lean and obese Spontaneously hypertensive rat/N-corpulent (SHR/N-cp) rats (7–8 weeks old) ⋅ Basal diet: American Institute of Nutrition 93G formula ⋅ Soy isoflavone (ISO) supplementation (0.1% *w*/*w*) ⋅ 20-week study	⋅ Tissue weights (peri-renal, ileal, subdiaphragmatic, epididymal fat pads)	⋅ Soy ISO ↓ body weight of obese rats ⋅ Soy ISO ↓ peri-renal, epididymal, and subdiaphragmatic fat pad weights in lean and obese rats ⋅ Soy ISO ↓ ileal fat pads in obese rats
	Zanella et al. [70]	⋅ Male C57BL6/J mice (3-weeks old) ⋅ Basal diet: low-fat diet (LFD) ⋅ Study 1 groups: (1) LFD (soy-free); (2) LFD + Soybean supplementation (8.5% *w*/*w*) ⋅ Study 2 groups: (1) LFD + vehicle (sham control); (2) LFD + genistein (5 mg/kg/day by oral gavage) ⋅ Study 1: 147 days; Study 2: 15 days	⋅ Glucose tolerance test (glucometer) ⋅ Insulin resistance test (0.75 Units insulin injected intraperitoneally; tail vein blood samples) ⋅ Body composition (Echo magnetic resonance imaging) ⋅ Tissue weights (epididymal and peri-renal fat pads)	⋅ Soy treatment ↑ total fat mass and ↑ fat pad mass vs. soy-free control group ⋅ Genistein ↑ adipose tissue mass vs. sham control ⋅ No differences in lean mass or total body weight between groups in Study 1 or Study 2 ⋅ No difference in glucose metabolism or insulin sensitivity between groups in Study 1 or Study 2
	Kavanagh et al. [80]	⋅ Mature insulin-resistant female monkeys (cynomolgus macaques and African green monkeys) ⋅ Soy isoflavone (ISO) supplementation (155 mg/day) ⋅ 4-month study	⋅ Glucose tolerance test (femoral intravenous collection: 5, 10, 20, 30, 60 min) ⋅ Insulin resistance test (hyperinsulinemic-euglycemic clamp) ⋅ Fasting plasma glucose (colorimetric assay) ⋅ Fasting insulin (ELISA kit) ⋅ Fasting plasma adiponectin (ELISA kit)	⋅ ISO ↑ insulin area under the curve vs. control ⋅ No effect of ISO on glucose area under the curve ⋅ No effect of ISO on circulating adiponectin levels
	Zhou et al. [86]	⋅ Huangjiang mini-piglets (weaned at 21 days of age) ⋅ Groups: (1) Basal diet + 0.5% *w*/*w* corn starch (control); (2) Basal diet + 0.5% *w*/*w* soybean galactooligosaccharides (SBOS) ⋅ 14-day study	⋅ Short-chain fatty acid analysis (intestinal luminal samples by gas chromatography) ⋅ Gut microbial composition (RT-quantitative PCR)	⋅ SBOS ↑ intestinal short-chain fatty acids and number of beneficial bacteria vs. control group ⋅ SBOS ↓ numbers of harmful bacteria vs. control group
Chickpea (*Cicer arietinum* L.)	Dai et al. [41]	⋅ Male CD-1 (ICR) IGS mice (6-weeks old) ⋅ α-galactooligosaccharides (α-GOS) extracted from chickpeas added to drinking water ⋅ Groups: (1) Normal chow; (2) HFD; (3) HFD + low-dose α-GOS (0.083 g/kg/day); (4) HFD + medium-dose α-GOS (0.42 g/kg/day); (5) HFD + high-dose α-GOS (0.83 g/kg/day) ⋅ 6-week study	⋅ Blood glucose (glucometer) ⋅ Serum insulin (rat/mouse insulin enzyme-linked immunosorbent assay (ELISA) kit) ⋅ Insulin resistance index (HOMA-IR) ⋅ Short-chain fatty acid analysis (luminal samples by high-performance liquid chromatography) ⋅ Gut microbial composition (quantitative PCR of fresh fecal samples)	⋅ HFD ↓ short-chain fatty acids, total bacterial quantity, & altered microbial composition ⋅ α-GOS ↑ short-chain fatty acids vs. HFD and normal chow groups ⋅ α-GOS stimulated Bifidobacterium and Lactobacillus growth vs. HFD
	Yang et al. [66]	⋅ Male Sprague Dawley rats (8 weeks old) ⋅ Diets: (1) standard chow; (2) high-fat diet (HFD); (3) HFD + chickpeas (10% raw, crushed chickpea seeds) ⋅ 8-month study	⋅ Insulin tolerance test (insulin at 2 Units/kg, intraperitoneal) ⋅ Oral glucose tolerance test (fasting glucometer readings from tail vein) ⋅ Fasting serum leptin (rat leptin radioimmunoassay (RIA) kit) ⋅ Fasting serum insulin (rat insulin ELISA kit) ⋅ Tissue weights (epididymal fat, skeletal muscle, liver) ⋅ Triglyceride content (Folch extraction method) ⋅ mRNA levels (Northern blot analysis of total RNA)	⋅ Chickpeas ↓ HOMA-IR, postprandial glucose, postprandial insulin levels vs. HFD ⋅ Chickpeas ↓ epididymal fat pad weight vs. HFD ⋅ Chickpeas ↓ hepatic and muscle triglycerides vs. HFD ⋅ Chickpeas ↓ leptin mRNA levels vs. HFD in epididymal adipose
N/A	Choi et al. [65]	⋅ Female Sprague Dawley rats (5 weeks old) ⋅ Ovariectomy (OVX) or sham operation ⋅ Groups: (1) Sham + HFD; (2) OVX + HFD; (3) OVX + HFD + genistein (0.1% *w*/*w* ; 90% pure) ⋅ 4-week study	⋅ Fasting serum glucose (enzyme assay kit) ⋅ Fasting serum insulin, adiponectin, leptin (ELISA kits) ⋅ Insulin sensitivity (HOMA-IR) ⋅ Tissue weights (liver and adipose) ⋅ Adipocyte histology (hematoxylin & eosin stain) ⋅ Gene expression (microarray analysis)	⋅ Genistein ↓ HOMA-IR vs. OVX group fed HFD ⋅ Genistein ↓ triglyceride accumulation and gonadal fat pad ewight ⋅ Genistein ↓ hepatic fatty acid synthase activity ⋅ Genistein ↓ fat synthesis genes ⋅ Genistein ↑ fat degradation genes ⋅ No differences in serum adiponectin or leptin between groups
N/A	Weitkunat et al. [84]	⋅ Male C57BL/6J mice (8 weeks old) ⋅ Diets: (1) LFD; (2) HFD with 10% dietary fibre (HFD control); (3) HFD with 5% SCFA and 5% dietary fibre ⋅ 30-week study	⋅ Oral glucose tolerance test (fasting glucometer readings from tail vein) ⋅ Insulin sensitivity (HOMA-IR) ⋅ Adipocyte histology (hematoxylin & eosin stain) ⋅ mRNA levels (RT-PCR) ⋅ Enzyme activity (developed assays)	⋅ SCFA ↓ HOMA-IR vs. HFD control group ⋅ Negative correlation between propionate and HOMA-IR ⋅ SCFA ↓ total adipocyte numbers and promoted small adipocytes vs. HFD control group ⋅ SCFA ↑ cytochrome c oxidase activity vs. HFD control group ⋅ SCFA ↑ mRNA levels of adipocyte browning markers
N/A	Stahel et al. [91]	⋅ Male Sprague Dawley rats (225–250 g) ⋅ Groups: (1) Basal diet + 15% methylcellulose (Control); (2) Basal diet + 15% *w*/*w* GOS ⋅ 9-week study	⋅ Insulin sensitivity (hyperinsulinemic-euglycemic clamp) ⋅ Gut microbial populations (DNA isolated from fecal samples)	⋅ GOS ↑ *Bifidobacterium* spp., Bacteroidetes, *Lactobacillus* spp. vs. control group ⋅ No differences in insulin sensitivity between GOS and control

Abbreviations: ↓, decrease; ↑, increase; ELISA, enzyme-linked immunosorbent assay; α-GOS, α-galactooligosaccharides; HFD, high-fat diet; HOMA-IR, homeostasis model assessment-insulin resistance; i.g., intragastric administration; IR, insulin resistant; ISO, soy isoflavones; mRNA, messenger ribonucleic acid; RIA, radioimmunoassay; RT-PCR, quantitative real-time polymerase chain reaction; SHR/N-cp, Spontaneously hypertensive rat/N-corpulent.

**Table 3:** Summary of the in vitro studies included in the present review.

Legume Source	Reference	Cell Line and Treatment	Experimental Methods	Key Findings
Chickpea (*Cicer arietinum* L.)	Gao et al. [32]	⋅ 3T3-L1 preadipocytes ⋅ Differentiated post-confluence ⋅ Control treatment (DMEM) ⋅ Isoflavones (ISO) extracted from chickpea seeds and sprouts (50 and 100 μg/mL) ⋅ Cells treated with chickpea isoflavones for 48 h	⋅ Isoflavone analysis (high-performance liquid chromatography (HPLC)) ⋅ Glucose uptake (using 2-deoxy-d-[^3^H]-glucose and glucose) ⋅ mRNA levels (quantitative real-time polymerase chain reaction (RT-PCR); 40 cycles) ⋅ Protein levels (Western blotting)	⋅ Chickpea ISO ↓ PPARγ and C/EBPα mRNA and protein levels vs. control in differentiated adipocytes ⋅ Chickpea ISO ↓ GLUT4 mRNA and protein levels vs. control in differentiated adipocytes
Soybean (*Glycine max.* L.)	Huang et al. [49]	⋅ 3T3-L1 preadipocytes ⋅ Differentiated post-confluence ⋅ Insulin resistance induced (1 mM dexamethasone, 10% fetal bovine serum, 10 mg/L insulin for 2 days) ⋅ Vehicle (0.1% dimethyl sulfoxide (DMSO)) ⋅ Black soybean koji (BSK) powder extract (25, 50, 100, 200 μg/mL) ⋅ BSK treatment for 60 h for preadipocytes ⋅ BSK treatment for 8 days for differentiating adipocytes	⋅ Isoflavone analysis (HPLC) ⋅ Glucose uptake (Benedict’s Test) ⋅ Protein levels (Western blotting)	⋅ BSK (200 μg/mL) ↑ glucose uptake vs. vehicle in preadipocytes ⋅ BSK (25–200 μg/mL) ↑ GLUT4 protein levels vs. vehicle in preadipocytes ⋅ BSK (50 to 200 μg/mL) ↑ adiponectin protein levels vs. vehicle in mature adipocytes ⋅ BSK (200 μg/mL) ↓ PPARγ protein levels vs. vehicle in preadipocytes
	Inaguma et al. [53]	⋅ 3T3-L1 adipocytes ⋅ Differentiated after 48 h; post-confluence ⋅ Cyanidin-3-glucoside from black soybeans (20 and 100 μM) added every 2 days for 4 days	⋅ Gene expression levels (RT-PCR; 40 cycles)	⋅ Cyanidin-3-glucoside ↑ GLUT4 mRNA levels vs. vehicle
	Kim et al. [60]	⋅ 3T3-L1 adipocytes ⋅ Differentiated post-confluence ⋅ Vehicle treatment (DMSO) ⋅ Black soybean anthocyanin extract (12.5 and 50 μg/mL) for 24 and 48 h	⋅ Anthocyanin extract analysis (HPLC) ⋅ Protein levels (Western blotting)	⋅ Anthocyanins (50 μg/mL) ↓ PPARγ protein levels vs. vehicle
N/A	Luna-Vital et al. [54]	⋅ 3T3-L1 adipocytes ⋅ Differentiated after 48 h ⋅ Insulin resistance induced (mTNFα 10 ng/mL for 6 days) ⋅ Anthocyanin-rich purple corn water extract (PCW; 0.4 mg dry sample/mL; exposure time unknown)	⋅ Insulin sensitivity measured via glucose uptake (2-NBDG assay)	⋅ PCW ↑ glucose uptake vs. vehicle in insulin resistant adipocytes

Abbreviations: ↓, decrease; ↑, increase; BSK, black soybean koji extract; C/EBPα, CCAAT-enhancer binding protein-alpha; DMSO, dimethyl sulfoxide; GLUT4, glucose transporter-4; HPLC, high-performance liquid chromatography; ISO, isoflavones; mRNA, messenger RNA; RT-PCR, quantitative real-time polymerase chain reaction; PPARγ, peroxisome proliferator-activated receptor-gamma.

**Table 4:** Summary of the proposed mechanisms of action of soybeans and chickpeas to improve insulin sensitivity.

Legume	Compound	Model	Proposed Mechanisms						
			GLUT4	PPAR-γ	Adiposity	Adiponectin	Leptin	Resistin	Gut Microbiota (SCFA-Producing Bacteria)
Black soybean	Isoflavones	3T3-L1 cells	↑ [49]	↓ [49]	↑ [49]	↑ [49]			
	Anthocyanins	3T3-L1 cells	↑ [53]	↓ [60]					
		Diabetic rats	↑ [38]						
Soybean	Isoflavones	Insulin resistant rats			↓ [1]	↑ [1]	↑ [1]	↓ [1]	
		Lean & obese rats			↓ [64]				
		C57BL/6 mice			↑ [70]				
		Insulin resistant, menopausal monkeys				X [80]			
	Galactooligosaccharides	Mini-piglets							↑ [86]
Chickpea	Isoflavones	3T3-L1 cells	↓ [32]	↓ [32]	↓ [32]				
	n/a	SD rats			↓ [66]		↓ [66]		
	α-galactooligosaccharides	CD-1 mice							↑ [41]

Abbreviations: ↑, increased effect; ↓, decreased effect; X, no effect; SCFA, short-chain fatty acid; SD, Sprague Dawley.
